# Supplementary figures and images for: Single-genome analysis reveals heterogeneous association of the Herpes Simplex Virus genome with H3K27me2 and the reader PHF20L1 following infection of human fibroblasts
Source: bioRxiv. 2023 Dec 3:2023.12.03.569766. Preprint. [Version 1] doi: 10.1101/2023.12.03.569766 (PMC10705572; doi:10.1101/2023.12.03.569766)

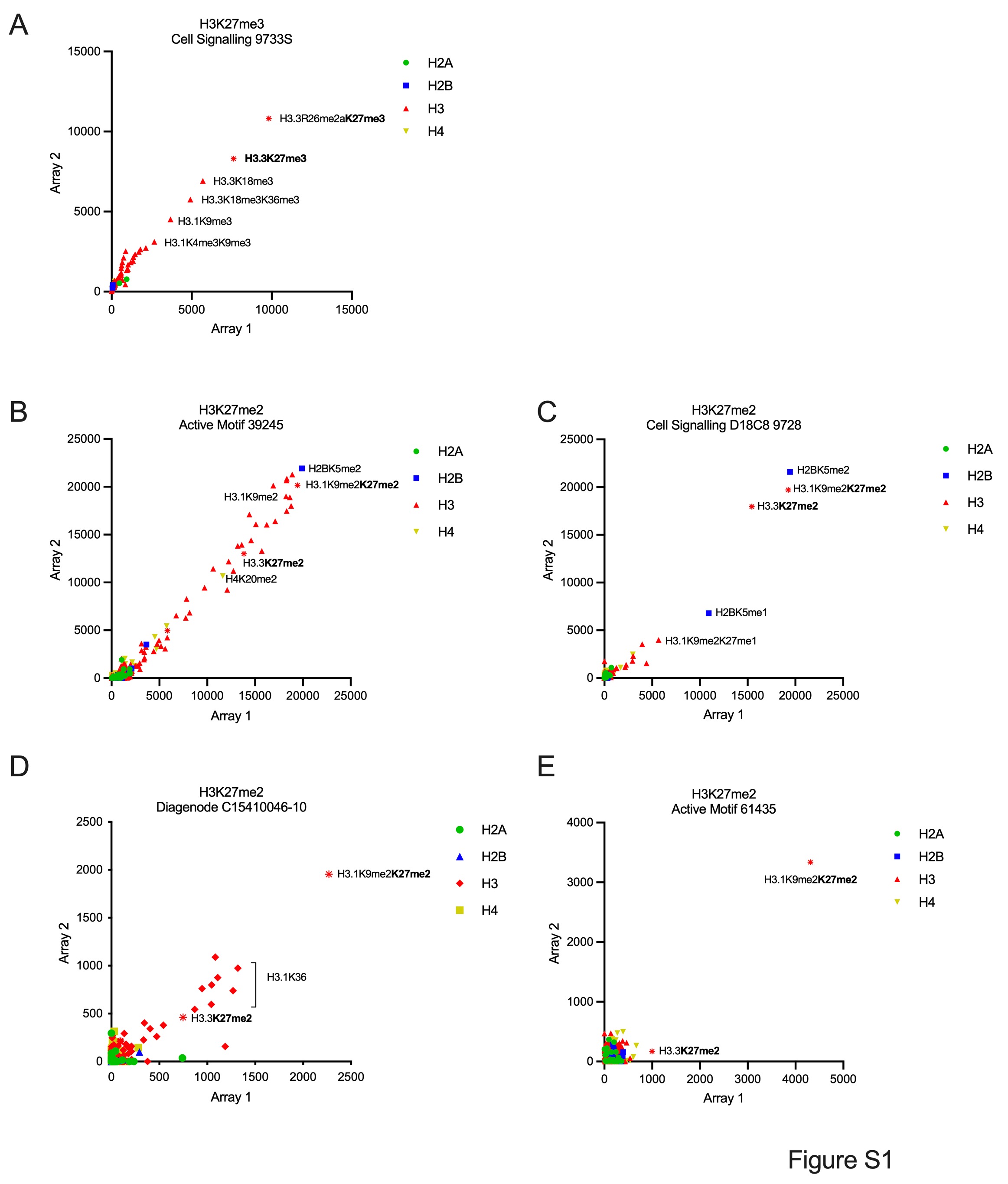

Supplement: Supplement 1 — Scatter plots of two binding array data sets from the same antibody sample, one dataset on each axis. Labels are bolded where the target residue is included. Other notable non-specific binding partners are also labeled. [file media-1.jpg]

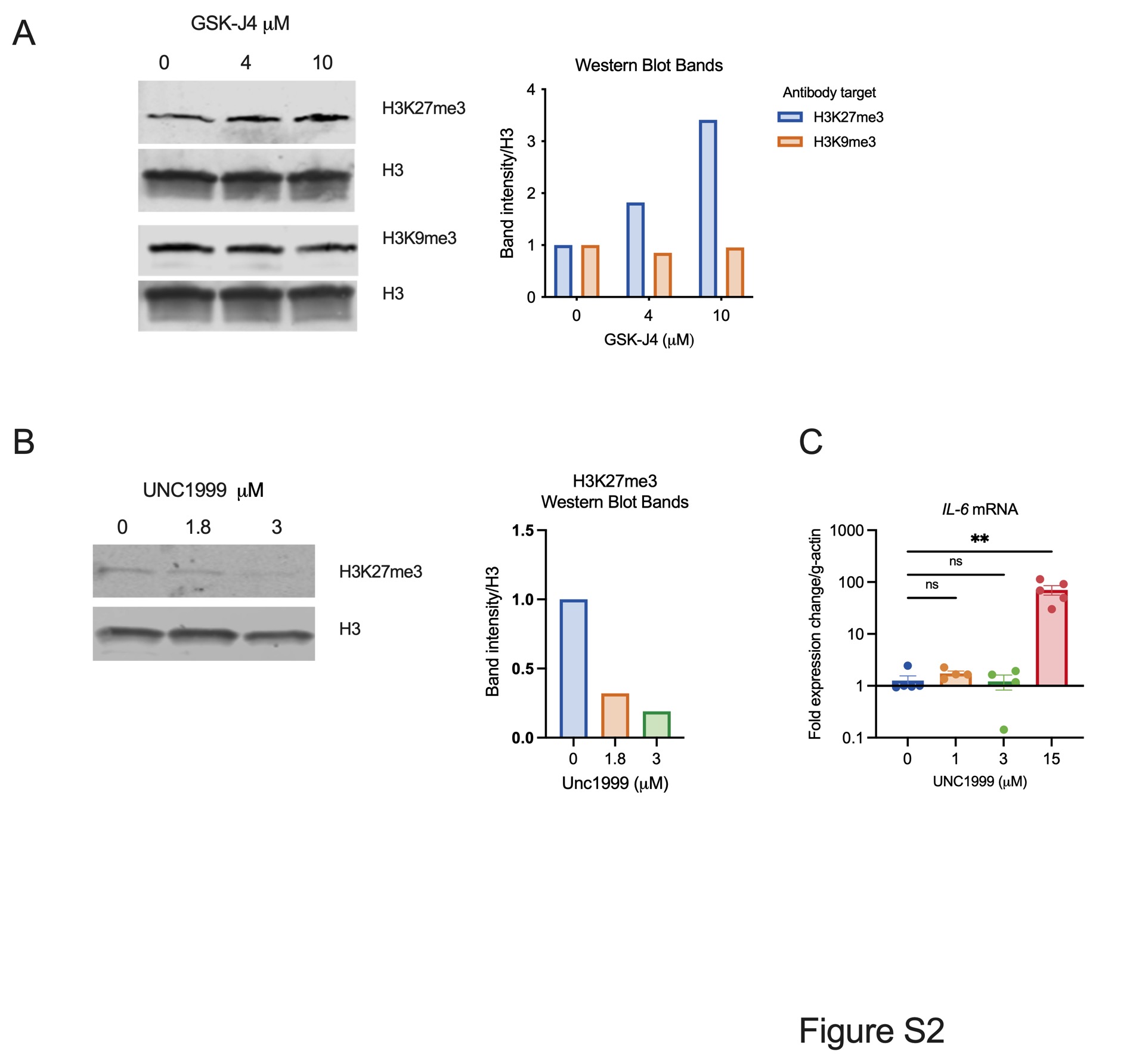

Supplement: Supplement 2 — (A) HFFs were treated with UNC1999 at indicated concentrations for 4 days, with fresh inhibitor added once on day 2. The cumulative effect of UNC1999 on cellular chromatin was assessed from histone extracts blotted for H3K27me3. Li-Cor band quantification is normalized to total H3 bands, relative to untreated cells. (B) Cumulative effects of treatment with GSK-J4 for four days on cellular chromatin, assessed by blotting histone extracts for both H3K27me3 and H3K9me3. Li-Cor band quantification in A and B was normalized to total H3 bands, relative to untreated cells. (C) IL-6 expression measured by RT-qPCR of cDNA made from HFFs treated with indicated concentrations of UNC1999 for 5 hours. [file media-2.jpg]

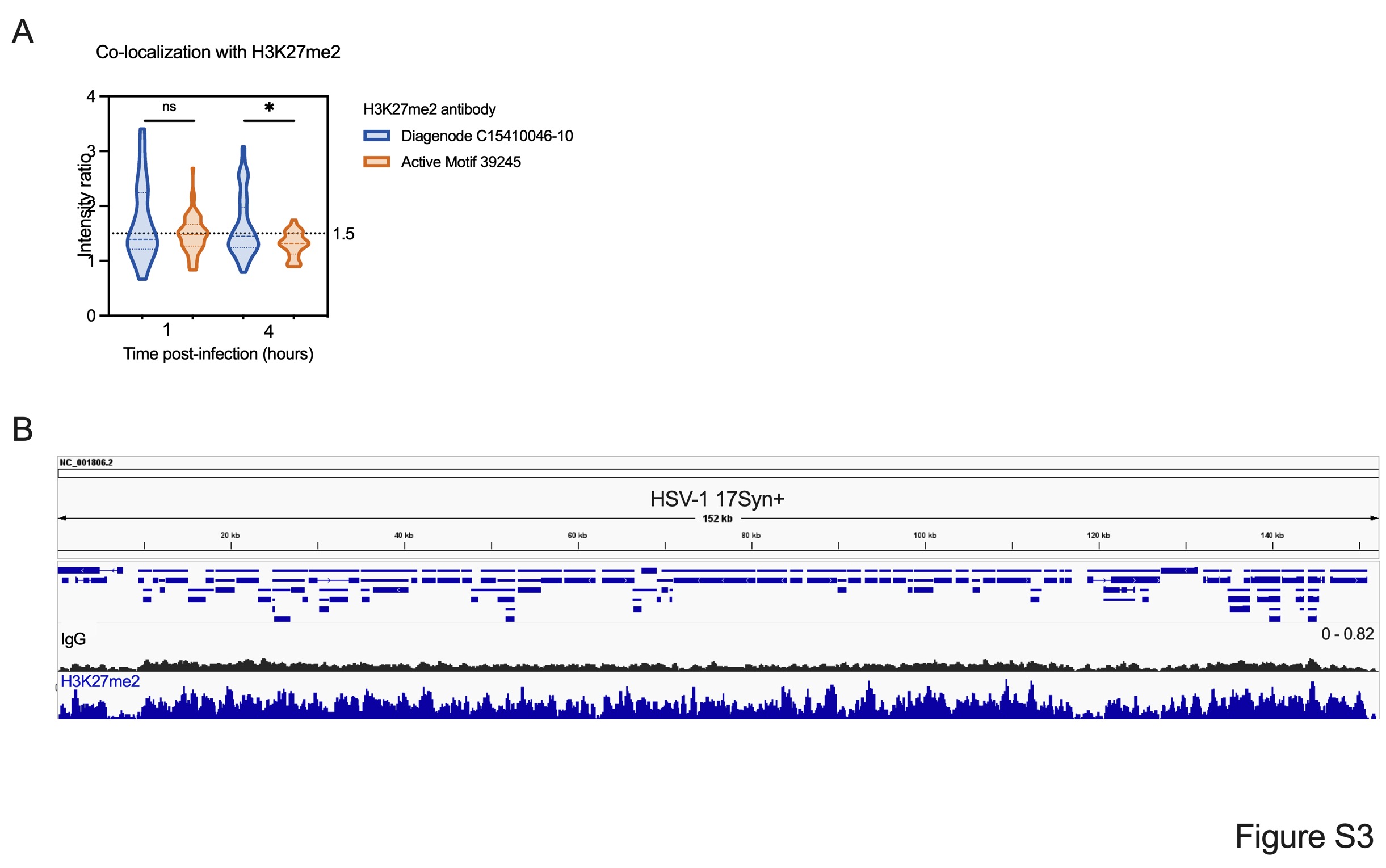

Supplement: Supplement 3 — (A) Comparison of co-localization with H3K27me2 immunostained with two different antibodies at 4 hpi (Kolmogorov-Smirnov test.) Adjusted p-value *=<0.05. (B) 17Syn+ genome coverage from HFFs 1 hpi, from a single replicate of CUT&RUN with control IgG and H3K27me2 antibodies (Diagenode C15410046-10). [file media-3.jpg]
